# A Look inside the *Listeria monocytogenes* Biofilms Extracellular Matrix

**DOI:** 10.3390/microorganisms4030022

**Published:** 2016-07-05

**Authors:** Angelo Colagiorgi, Pierluigi Di Ciccio, Emanuela Zanardi, Sergio Ghidini, Adriana Ianieri

**Affiliations:** Department of Food Science, University of Parma, Via del Taglio 10, Parma 43126, Italy; angelo.colagiorgi@studenti.unipr.it (A.C.); emanuela.zanardi@unipr.it (E.Z.); sergio.ghidini@unipr.it (S.G.); adriana.ianieri@unipr.it (A.I.)

**Keywords:** *Listeria monocytogenes*, listeriosis, extracellular polymeric substances (EPS’s), biofilm, extracellular matrix, foodborne pathogens, food poisoning

## Abstract

*Listeria monocytogenes* is a foodborne pathogen able to persist in food industry and is responsible for a severe illness called listeriosis. The ability of *L. monocytogenes* to persist in environments is due to its capacity to form biofilms that are a sessile community of microorganisms embedded in a self-produced matrix of extracellular polymeric substances (EPS’s). In this review, we summarized recent efforts performed in order to better characterize the polymeric substances that compose the extracellular matrix (ECM) of *L. monocytogenes* biofilms. EPS extraction and analysis led to the identification of polysaccharides, proteins, extracellular DNA, and other molecules within the listerial ECM. All this knowledge will be useful for increasing food protection, suggesting effective strategies for the minimization of persistence of *L. monocytogenes* in food industry environments.

## 1. Introduction

*Listeria monocytogenes* is an ubiquitous bacterium isolated from soil, water, wastewater, animals, humans, and raw or processed food [1]. It is a Gram-positive, facultative anaerobe, non-spore-forming, foodborne pathogen. *L. monocytogenes* is able to grow and survive in different environmental conditions due to its tolerance to low temperatures (4 °C–10 °C), high pH, and high salt concentrations [2], and the presence of polar flagella confers to such a microorganism a tumbling motility at room temperature [3].

**Figure 1:**
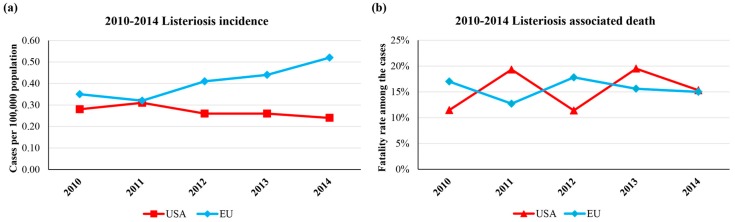
Listeriosis incidence and associated death in the USA and the EU from 2010 to 2014. Panel (**a**) shows the number of cases of listeriosis per 100,000 population; in panel (**b**), the percentage of listeriosis associated death among the confirmed illness cases are reported.

*L. monocytogenes* is the etiological agent of a severe infection called listeriosis [4]. Although the disease is rare, listeriosis is an important public health problem because it is associated with high hospitalization and mortality rates. According to European Food Security Authority (EFSA), 1300 cases of listeriosis occur annually in the European Union (EU), whereas the Centers for Disease Control and Prevention (CDC) estimates that approximately 1600 cases occur every year in the United States of America (USA) [5,6,7,8,9], with a statistically significant increasing trend of listeriosis in the 2008–2014 period in the EU [10,11] (Figure 1).

Elderly, pregnant women, newborns, and adults with weakened immune systems are the most affected by the disease, and septicemia, meningitis, miscarriage, and stillbirth are common clinical presentations [12,13,14], although people without these risk factors can also be affected [15,16].

The principal route of transmission is the consumption of contaminated food due to the ability of *L. monocytogenes* to survive and grow in acidic or salty conditions and to replicate at low temperatures [17,18], and to colonize food processing environments, including equipment used in food processing operations [19], representing a serious concern for human health [20]. *L. monocytogenes* attach to a variety of surfaces, including stainless steel, polystyrene, and glass [21,22,23], and are able to form biofilms. Microbial cells in biofilms are embedded in a self-produced matrix of extracellular material, composed by a conglomeration of different types of biopolymers known as extracellular polymeric substances (EPS’s) [24]. EPS’s form the scaffold for a three-dimensional structure of the biofilm and are responsible for the adhesion to surfaces and cohesion in the biofilm [25]. Several features of a biofilm may vary depending on the production, the quantity, and the characteristics of the individual components of EPS’s, such as cohesion, charge, concentration, and nature of molecules [26].

The biofilm matrix can account for more than 90% of the dry mass of a biofilm [25]. Polysaccharides, proteins, and DNA compose the extracellular matrix of many biofilms [25]. These molecules are involved in adhesion to surface, cohesion within the biofilm, and aggregation of bacterial cells. Furthermore, retention of water (preventing desiccation), protection against antimicrobial agents (acting as protective barrier), sorption of organic and inorganic compounds (acting as a reservoir of energy), and enzymatic activity are some of the activities performed by EPS’s in bacterial biofilms [25].

Many studies have been carried out aiming to better understand the complexity of the biofilm extracellular material in both Gram-positive and -negative microorganisms. Several molecules have been identified and characterized, i.e., different exopolysaccharides, proteins and extracellular DNA (eDNA), in the biofilm matrix of *Staphylococcus aureus*, *Escherichia coli*, *Pseudomonas aeruginosa*, *Vibrio cholerae*, and others [27,28,29,30]. However, as for *L. monocytogenes*, information is so far not abundant. In this review, we summarize the status of experimental efforts aimed at characterizing ECM of *L. monocytogenes* biofilms.

## 2. *Listeria monocytogenes* and Biofilms

*L. monocytogenes* is able to persist in food environments, representing an important source of contamination that can lead to food spoilage or transmission of disease [22]. Biofilm formation is important for the survival of *L. monocytogenes* in the food industry, e.g., it was observed that biofilms formed by strains that persist in the industry are thicker than those formed by isolates found only sporadically [31]. Furthermore, listerial cells embedded in biofilms are different in structure and physiology from the planktonic ones, e.g., adherent cells change their shape from rod to coccoid as the population aged, grew more slowly [32], are more resistant to antibiotics and sanitizers, and harder to remove and destroy [33,34]. The ability to form biofilms has been reported to be different among different isolates, but it was not possible to establish a clear correlation with serovars or lineages [35,36]. Many authors have agreed that *L. monocytogenes* biofilms are strongly influenced by temperature [22,37,38], strain [35,39,40], incubation time [41], medium [37,38], and the nature of the adhesion surface [42,43]. Moreover, the fatty acids profile of biofilm forming listerial cells was analyzed, and an increase in the amount of certain fatty acids (iso-C_14:0_, anteiso-C_15:0_, and iso-C_16:0_) with an increase in biofilm forming ability of the isolates was observed, possibly indicating a relationship between total fatty acid composition of the biofilm and the biofilm-forming ability of a strain [44,45,46].

A variety of structures of *L. monocytogenes* biofilms have been described, including a monolayer of adherent cells, flat unstructured multilayers, and a knitted-chain network, depending on the strains and experimental setup used [35,39,47,48,49,50]. Recently, Guilbaud et al. [51], using three-dimensional reconstruction of confocal laser scanning microscopy (CLSM) images acquired by Leica SP2 AOBS Confocal Laser Scanning Microscope (Leica-Microsystems, Wetzlar, Germany), observed *L. monocytogenes* intraspecies diversity in the ability to form biofilm. They analyzed biofilm architecture formed by 96 isolates and observed that the majority of listerial strains formed honeycomb-like structures consisting of layers of cohesive cells, heterogeneously distributed, decorated with hollow voids with diameters ranging from 5 to 50 μm. Within the hollow voids, swimming bacteria were observed, whereas an inner compact matrix was lacking. Biofilm staining with propidium iodide revealed pockets formed by dead cells and eDNA. This spatial organization is common to various microorganisms, since it has previously been described in staphylococcal species [28,52] and in *Enterococcus faecalis* [53], and has been reported for *L. monocytogenes* by Marsh et al. [49].

In the end, it appears that a multitude of parameters influence the biofilm structure of *L. monocytogenes*, such as the strain, the surface, and other environmental conditions, such as pH, temperature, and medium [47].

## 3. The *L. monocytogenes* Biofilm Extracellular Matrix

Since the ability of *L. monocytogenes* to produce EPS’s has been controversial for a long time, several studies have been done in order to demonstrate the extracellular matrix production by various listerial strains. Staining with ruthenium red [35,54] and Congo red [55,56], fluorescein-conjugated lectin binding [57], fluorescent dye-conjugated antibody binding [58], phenolic sulfuric acid analysis [59], and fibril or matrix analysis via scanning electron microscope (SEM) [35,54,57,59] have been performed. Extracellular matrix composition has been examined mainly by nuclear magnetic resonance (NMR, Bruker, Billerica, Massachusetts, MA, USA) [60,61], gas chromatography-mass spectrometry (GC-MS, Thermo Scientific, Waltham, Massachusetts, MA, USA) [60], and CLSM [51,62] analyses. Here, we report the current knowledge about *Listeria* biofilm extracellular matrix, as summarized in Table 1.

### 3.1. Exopolysaccharides and Teichoic Acids

Exopolysaccharides are the major components of the extracellular matrix in many microorganisms [63,64]. In mucoid strains of *Pseudomonas aeruginosa*, alginate is the principal component of EPS’s [65], whereas poly-*N*-acetyl glucosamine (PNAG) and teichoic acids (TAs) were found in the *S. aureus* biofilm matrix [66]. In the early 2000s, Borucki et al. [35] reported the presence of carbohydrates in all *L. monocytogenes* strains tested in their work, with the highest biofilm formers producing noticeably more polysaccharides [35]. Recent studies have deeply investigated the structure of the carbohydrate component of the *L. monocytogenes* extracellular matrix.

As reported recently, Alonso et al. [67] performed a transposon mutagenesis library screen using a Himar1 *mariner* transposon in order to investigate genetic elements involved in biofilm formation. The authors identified a total of 38 genetic loci involved in the biofilm formation process. In particular, they investigated further two of these loci, the d-alanylation pathway genes *dltABCD* and the phosphate-sensing two-component system *phoPR*. The results, obtained by deletion of these loci, indicated a significant reduction in biofilm formation by the mutant strains compared with wild-type bacteria in a polyvinyl chloride (PVC) microtiter plate assay and by CLSM. The d-alanylation of lipoteichoic acids (LTAs) and the phosphate-sensing *phoPR* two-component system both appear to play critical roles for biofilm formation by *L. monocytogenes.*

The importance of TAs within the *L. monocytogenes* biofilm matrix was confirmed by Brauge et al. [60]. GC-MS (Thermo Scientific, Waltham, Massachusetts, MA, USA) and ^13^C- and ^1^H-NMR spectroscopy (Bruker, Billerica, Massachusetts, MA, USA) analyses of soluble carbohydrates extracted from a 48-h biofilm extracellular matrix and culture supernatant of six strains of *L. monocytogenes* led to the identification of TAs. Interestingly, such molecules were structurally identical to TAs isolated from the cell wall. Furthermore, a mutant strain encoding TAs lacking NAG glycosylation presented an extracellular carbohydrate fraction identical to the modified cell wall molecules. These findings suggest that extracellular and cell wall TAs may have the same origin, probably deriving from autolysis and the peeling of bacteria. Similar data were reported by Savdovskaya et al. [76] who compared extracellular and cell wall TA structures of the reference biofilm-positive strain *S. epidermidis* RP62A.

The extracellular TAs were the only carbohydrate polymers identified in the work of Brauge et al. [60], whereas Köseoğlu et al. [61] recently reported the presence of an insoluble cell-bound poly-β-(1,4)-*N*-acetylmannosamine decorated with terminal α-1,6-linked galactose (Man*N*Ac-Gal) identified in a constructed aggregate-forming mutant strain of EGD-e. These contrasting results may be explained considering that the native Man*N*Ac-Gal is completely insoluble, so the liquid-state NMR analysis by Köseoğlu et al. [61] on the listerial EPS’s was conducted after the *N*-deacetylation of such molecules. The biosynthesis of such EPS’s, produced by the *pssA-E* operon, is activated by elevated levels of the second messenger c-di-GMP, as previously observed in proteobacteria [77]. This molecule was found to be involved in biofilm formation and regulation in several bacterial species, i.e., regulation of alginate [78] and the glucose rich matrix polysaccharide Pel [79] biosynthesis in *P. aeruginosa*, and cellulose synthesis in many proteobacteria [80,81,82]. The c-di-GMP signaling networks are likely very complex, as a large number of c-di-GMP signaling proteins has been predicted in many bacterial species, and it is not clear how several input signals are integrated by microorganisms to control bacterial behavior [83,84]. Generally, high levels of c-di-GMP are required for bacteria to become sessile. The c-di-GMP-induced Man*N*Ac-Gal of *L. monocytogenes* was found to be responsible for various phenotypic traits, i.e., cell aggregation, reduced motility in semi-solid media, moderate decrease of invasiveness in mammalian cells, and a highly increased tolerance to disinfectants and dehydration, aiding bacteria to persist in the environment.

### 3.2. Extracellular and Biofilm-Associated Surface Proteins

The role of surface or extracellular proteins in the initial attachment of *L. monocytogenes* to a surface has been demonstrated in different studies [68,69,85]. In 2012, Combrouse et al. [59] firstly quantified the extracellular components of the *L. monocytogenes* biofilm and reported that extracellular proteins are the most abundant exopolymers within the listerial biofilm matrix. According to Longhi et al. [68], the protease treatment of listerial biofilm is able to impair biofilm development or to induce dispersal of the cells. Moreover, Nguyen et al. [69] recently observed the reduction of established biofilms to undetectable levels after treatment with proteinase K. Treatment with proteinase K led to a noticeable increase in planktonic cell density. According to other findings, the protease inhibitor α2-macroglobulin severely impairs biofilm formation, suggesting that proteolytically processed proteins are more likely to be part of the *L. monocytogenes* biofilms [70]. All these results suggest that proteins within the biofilm matrix or on the cell surface are a key part of the EPS matrix. Nonetheless, there is a paucity of information about the characterization of the proteic components of the listerial matrix.

*L. monocytogenes* is able to encode more than 130 surface proteins that can confer to this bacterium the ability to survive in diverse environments [86,87]. Among these, Internalin A (InlA) is a cell-wall-bound protein and is one of the major components involved in the adhesion to and invasion of a host cell expressing a specific form of E-cadherin. Franciosa et al. [70] found that *L. monocytogenes* strains encoding a truncated non-functional form of the InlA protein exhibited significantly enhanced biofilm-forming ability compared with wild-type strains. Such a truncated molecule, fully released in the extracellular medium, was hypothesized to be part of the biofilm matrix.

Biofilm-associated protein (Bap) is a surface protein involved in biofilm formation in different bacterial species [88], such as *S. aureus, Enterococcus faecalis*, or the Gram-negative *Salmonella enterica* sv typhimurium [89,90,91,92]. The high-molecular-weight Bap-related proteins present a core domain of tandem repeats and are able to confer to bacteria the capacity to form a biofilm [88]. Concerning *L. monocytogenes*, Jordan et al. [71] reported the presence of a protein similar to Bap, presenting Bap-like structural features and thus designated BapL. Similar to some Bap-related proteins found in other species, such a molecule is required for cell attachment to abiotic surfaces, while on the contrary, it is not required for virulence [71]. BapL can contribute to the attachment of some *L. monocytogenes* strains, but its role in biofilm development has not been clearly established: Renier et al. [47], in fact, showed that some BapL-negative strains were able to adhere significantly better than BapL-positive strains, whereas the attachment ability of other strains was weakened.

Comparison of exoproteome of *L. monocytogenes* from biofilm and planktonic state by Lourenço et al. [73] led to the identification of proteins in higher abundance in the biofilm exoproteome. Phospholipase PlcA, flagellin (FlaA), a putative penicillin-binding protein (PBP), an actin assembly inducing protein (ActA), and a putative cell wall binding protein (Lmo2504) were among these identified proteins. Flagellin is a monomer that polymerizes to form the flagella. Different groups reported the importance of flagella during *L. monocytogenes* biofilm development [72,93], but their exact role in the process remains unclear because of conflicting results. It is well-known that *L. monocytogenes* has a temperature-dependent motility, since flagellin is expressed between 20 and 25 °C [94]. According to Lourenço et al. [73] and Hefford et al. [57], the amount of flagellin within the proteome is higher in biofilms than in planktonic bacteria, although Trémoulet et al. [32] reported contrasting findings. Lemon et al. [72] found that flagellum-minus and paralyzed-flagellum mutants were defective in the step of initial attachment to surface and in subsequent biofilm formation. According to their findings, the importance of flagella in biofilm formation is related to their role in motility of cells, since the ability to form a biofilm of different flagella mutant strains was restored by supplying surface-directed motility exogenously via centrifugation. According to Zetzmann et al. [62], listerial flagella could have a role in biofilm formation under a static condition, whereas flagellar motility may have the opposite effect when bacteria are in a flow chamber. SEM observations by Guilbaud et al. [51] recently revealed the presence of extracellular fibrils in the *L. monocytogenes* biofilm honeycomb-like structures, putatively identified as flagella since they were absent in biofilm produced by non-motile or flagella mutant *L. monocytogenes* strains. Although biofilm formation was not prevented, the lack of such fibrils resulted in a flat and unstructured architecture of biofilm, suggesting a structural role in the three-dimensional architecture of listerial biofilms. It is still not clear whether either the flagella presence on its own or the flagellar motility are involved in biofilm formation and development; nonetheless, all findings suggest the importance of these structures during attachment to surface.

### 3.3. Extracellular DNA

Extracellular DNA is an important structural component of the EPS matrix of a wide range of Gram-positive and -negative bacteria [75], where it cooperates with proteins and polysaccharides ensuring structural integrity of the biofilm [95,96,97,98,99]. Concerning *L. monocytogenes*, Harmsen et al. [74] found the presence of DNA in stationary-phase cultures grown in a brain–heart-infusion (BHI) medium. They also showed that DNaseI treatment of the supernatant inhibited the initial attachment of bacteria to glass (in cultures diluted in PBS) and the delayed biofilm formation of bacteria grown in a minimal medium in polystyrene microtiter plates. Moreover, Zetzmann et al. [62] recently provided evidence that eDNA serves as a structural component of the EPS matrix of *L. monocytogenes* EGD-e, serotype 1/2a, in a diluted BHI medium under both static and dynamic conditions, suggesting the targeting of this molecule for preventing or dispersing biofilm formation of these microorganisms.

Different studies on eDNA of *L. monocytogenes* biofilms have been done for a better understanding of the origin of the nucleic acids in the extracellular matrix [62,74,75,100]. It has been hypothesized that, under environmental conditions, eDNA released by lysed bacteria (or present in the environment) supports an initial attachment to surfaces. The lysis of bacteria cells already attached to the surface could be increased by hypotonic conditions, and the released DNA may have a double role in the biofilm: to constitute an anchoring site for dividing cells in microcolonies and to serve as a scavenger for further planktonic cells recruiting [62]. The hypothesis that eDNA can take origin from lysed cells was already supported by Harmsen et al., who demonstrated the chromosomal origin of eDNA by polymerase chain reaction (PCR) amplification [74].

Besides its structural role, eDNA serves also as an energy and nutrition source. In the case of *L. monocytogenes*, Guibaud et al. [51] observed the presence of DNA pockets in biofilms with honeycomb-like structures, hypothesizing that this phenomenon could provide nutrients for the starved surviving subpopulation. Furthermore, eDNA represents a repertoire of genes from which naturally competent bacteria can derive genetic information through horizontal gene transfer (HGT), a mechanism by which genetic information is passed from one bacterial genome to another [74].

## 4. Conclusions and Perspectives

*L. monocytogenes* is an important foodborne pathogen thanks to its ability to persist in the food industry by the formation of biofilms. Several efforts have been made in order to better understand *L. monocytogenes* biofilm composition. The extracellular matrix is the major component of the biofilms and the most difficult to study due to the presence of a large range of biopolymers that are difficult to analyze. For this reason, it has also been called “the dark matter of biofilm” [101].

**Figure 2:**
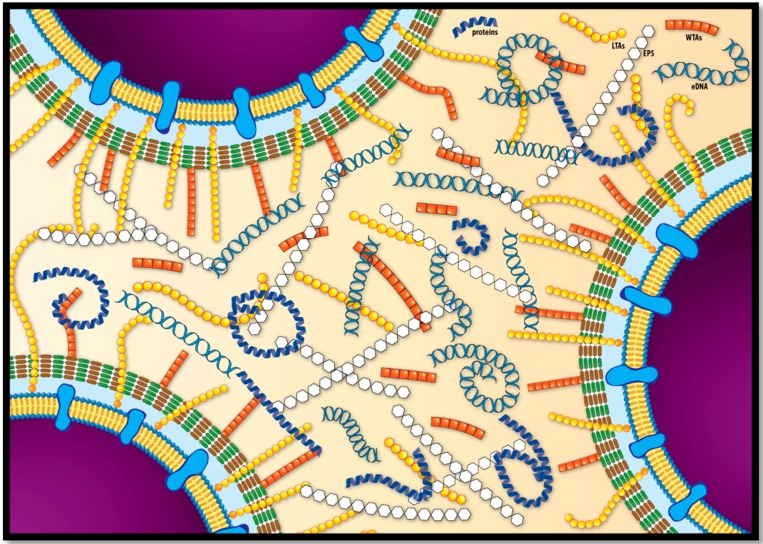
Schematic representation of listerial extracellular biofilm matrix. The major components (EPS’s, WTAs, LTAs, proteins, and eDNA) are distributed heterogeneously within the matrix.

The recent molecular and chemical studies focused on EPS matrix composition of *L. monocytogenes* have expanded the knowledge about biofilm structure of this pathogen. Several molecules have been characterized within the extracellular matrix, such as TAs, EPS’s, surface-associated proteins, flagella, and eDNA (Figure 2). Different studies have contributed to determining the complexity and the function of these components during biofilm formation and development, from the initial adhesion to surface to the dispersal of cells to the planktonic state. However, more efforts and studies are required for discovering and improving our understanding of the molecules and mechanisms involved in this process. A deeper knowledge of listerial biofilm composition and formation process is pivotal for developing effective strategies aimed at minimizing *L. monocytogenes* persistence in the food industry and therefore new foodborne outbreaks involving this pathogen.

## Figures and Tables

**Table 1:** Structural components of *Listeria monocytogenes* extracellular matrix. EPS’s characterized so far and their role within biofilm matrix are listed here.

EPS	Molecules	Features	References
**Polysaccharides**	poly-β-(1,4)-*N*-acetylmannosamine (poly-NAM) Teichoic Acids (WTA and LTA)	- Teichoic acids are the major component of listerial biofilm matrix	[60]
		- *dltABCD* mutants present a reduction in biofilm forming ability	[67]
		- Biosynthesis of poly-NAM is activated by high levels of c-di-GMP	[61]
**Proteins**	InlA BapL PlcA FlaA PBP ActA Lmo2504	- Proteinase K treatment impairs biofilm development, suggesting protein involvement in initial attachment	[68,69]
		- Truncated proteins exhibited enhanced biofilm forming ability	[70]
		- BapL can contribute to the attachment of some *L. monocytogenes* strains	[71]
		- Flagellar motility has controversial role in biofilm formation	[32,51,72,73]
**Extracellular DNA**		- DNAseI treatment inhibited or delayed initial attachment of bacteria to surfaces, suggesting eDNA involvement in initial attachment	[69,74]
		- Ensure structural integrity of biofilm in cooperation with proteins and polysaccharides	[62,74,75]
		- Involved in Horizontal Gene Transfer	[74]
		- Serves as energy and nutrition source	[51]
		- eDNA is released by lysed bacteria	[62,74]

NOTE: WTA: Wall Teichoic Acids; LTA: Lipoteichoic Acids; c-di-GMP: cyclic diguanosine monophosphate.

## Abbreviations

The following abbreviations are used in this manuscript:

BHI	brain heart infusion
CDC	Centers for Disease Control and Prevention
c-di-GMP	Cylic diguanosine monophosphate
CLSM	confocal laser scanning microscopy
ECM	extracellular matrix
EFSA	European Food Safety Authority
EPS’s	extracellular polymeric substances
GS-MS	gas chromatography-mass spectrometry
HGT	horizontal gene transfer
*L. monocytogenes*	*Listeria monocytogenes*
LTAs	lipoteichoic acids
NMR	nuclear magnetic resonance
PVC	polyvinyl chloride
SEM	scanning electron microscopy
TAs	teichoic acids
WTAs	wall teichoic acids
